# Identity and uncertainty: art-mediated medical student reflections in a time of transition

**DOI:** 10.1080/10872981.2022.2120946

**Published:** 2022-09-06

**Authors:** Talia Robledo-Gil, Elizabeth Ryznar, Margaret S. Chisolm, Kamna S. Balhara

**Affiliations:** a General Internal Medicine, Johns Hopkins University School of Medicine, MD, Baltimore, USA; b Psychiatry and Behavioral Sciences, Johns Hopkins University School of Medicine, Baltimore, Maryland, USA; c Psychiatry and Behavioral Sciences, and Professor of Medicine, Johns Hopkins University School of Medicine, Baltimore, Maryland, USA; d Department of Emergency Medicine, Johns Hopkins University School of Medicine, Baltimore, Maryland, USA

**Keywords:** Reflection, identity formation, professional development, art, medical training

## Abstract

Medical education comprises intense periods of transition, which can significantly impact student well-being, as well as personal and professional development. In 2020, medical students navigating transitions from pre-clinical to clinical roles were also experiencing the historic forces of the COVID-19 pandemic and ongoing societal reckoning with systemic injustice and racism, likely heightening the usual challenges associated with these transitions. Reflection has been suggested as a tool for facilitating such transitions, and arts-mediated approaches hold promise in inspiring authentic reflection, yet they are rarely used to prompt medical student reflection. This article describes common themes in medical students’ reflections on a specific period of transition during a unique moment in history, via qualitative analysis of their narrative responses to visual arts-mediated reflective prompts. The authors used a visual arts-based activity to explore medical students’ hopes and concerns as they transitioned to clinical clerkships between the 2019–2020 and 2020–2021 academic years at one academic institution. Qualitative analysis using an exploratory constructivist approach revealed that students’ reflections often focused on identity within three main themes: the personal self, the professional self, and the social self. Within these categories, subthemes included uncertainty and concerns focusing on medical training and knowledge, the sense of hope and value inherent to their social connections, critiques of the culture of medical education, and reflections on complicity and responsibility in racial injustice. This article not only provides a cross-sectional snapshot of the experiences of medical students during a historic moment, but also provides themes to guide discussions on training transitions and describes a low-cost, adaptable approach to facilitating deep exploration and reflection on tumultuous moments in training.

## Introduction

Transitions in medical education represent potentially tumultuous periods for medical students. The shift from pre-clinical classroom-based roles to clinical responsibilities in clerkships has been described as particularly challenging, with changes in learning environments; increases in workload leading to perceived insufficiency of knowledge; and ambiguity about roles contributing to feelings of inadequacy, anxiety, and stress [1,2]. The unique logistic and emotional upheaval from the COVID-19 pandemic and the ongoing societal reckoning with systemic injustice and racism in 2020 have likely heightened the usual challenges associated with these transitions.

Activities that encourage reflection, such as written narratives or shared group discussions, have been suggested as a means to process and understand medical education and training transitions [3–5]. Analyses of student narratives have examined how medical students navigate such professionally transformative moments [6,7], usually relying upon written prompts to guide students’ narrative reflections. A core component of these exercises is the creation of a ‘safe space’ [3]. Visual arts-mediated approaches have the potential to provide a relatively efficient way to develop the psychological safety necessary for authentic critical reflection. Works of art have been successfully used in medical education as a ‘third thing’ – a reflective trigger or conversational mediator that creates a safe space for personal reflection and sharing of diverse perspectives on potentially challenging topics, such as adverse events in patient care [8–10]. Traditional narrative reflection activities, including those relying on recall of specific positive or negative experiences, may be inadequate to generate authentic reflection [4]. The use of works of art and subsequent narrative metaphors accessed through images may help overcome certain barriers to such reflection (e.g., traditional teacher-learner hierarchies, fear of embarrassment) [8]. However, the use of visual art as a prompt for medical student reflections on moments of transitions remains relatively rare [11].

Given the additional personal and professional instability likely faced by medical students in 2020, and the relatively untapped potential of visual art as a means for reflection on key transitions, we sought to use a visual arts-based activity to explore medical students’ hopes and concerns as they transitioned to clinical clerkships between the 2019–2020 and 2020–2021 academic years. We identified common themes in medical students’ reflections on a specific period of transition during a unique moment in history, via qualitative analysis of their narrative responses to arts-mediated reflective prompts.

## Methods

We conducted a cross-sectional qualitative analysis of narrative reflections from a sample of third and fourth year students at one medical school in the USA. Students submitted these reflections as part of a session occurring during a multi-module virtual course required for all students prior to returning to or starting clinical clerkships, called Pre-Clerkship Education Exercises (PRECEDE). PRECEDE covers various components of preparing to enter clinical environments, and our session occurred during the ‘wellness, sleep, and surviving the wards’ module of the course. The medical school offered the PRECEDE course twice, in June and July 2020, both of which included our session.

As part of this session, students participated in a one-hour visual arts-mediated discussion – focused on maximizing well-being and resilience. Prior to this session, students were instructed to independently complete a written reflection activity based on viewing works of art from an online virtual art gallery. In the written reflection activity, students were asked to reflect on their hopes and concerns for the upcoming academic year and then to select an image from the online gallery that spoke to that hope or concern. After reflecting and selecting an image, students then wrote a brief explanation as to why they chose that image and submitted their response on an online platform (Qualtrics). The online gallery was curated by two faculty clinicians (KB and MC) with advanced training and experience in art museum-based health professions education. The gallery contains images with diverse subjects, formats, and media, including traditional and contemporary portraiture, still life, abstract art, and photography. Both faculty members have experience with Visual Thinking Strategies (VTS) and the VTS online collection served as the source of images for this gallery [12].

In August 2020, after completion of both iterations of the course, we emailed all students who had enrolled in the course and participated in the pre-session activity to opt into a research study that would analyze their de-identified narrative reflections. Only reflections from students who consented to the study after the course ended were included in the analysis.

Two study team members who were not involved in the session (TRG, a resident at the time, and ER, a junior faculty member) thematically analyzed and iteratively coded the participants’ narrative reflections using framework analysis [13]. They created and defined codes, which were ultimately collated into a codebook agreed upon by both coders. The coders achieved greater than 85% agreement on first pass, and ultimately 100% after team discussion. A third team member (MC, a senior faculty member) resolved any discrepancies. We utilized an exploratory approach, applying a constructivist paradigm. We used Atlas.ti for initial coding and to facilitate analysis.

All authors involved in this study are clinicians of various specialties, including internal medicine, emergency medicine, and psychiatry.

The study was reviewed by the Johns Hopkins Institutional Review Board (IRB# 00257174) and deemed exempt.

## Results

**Figure 1.**
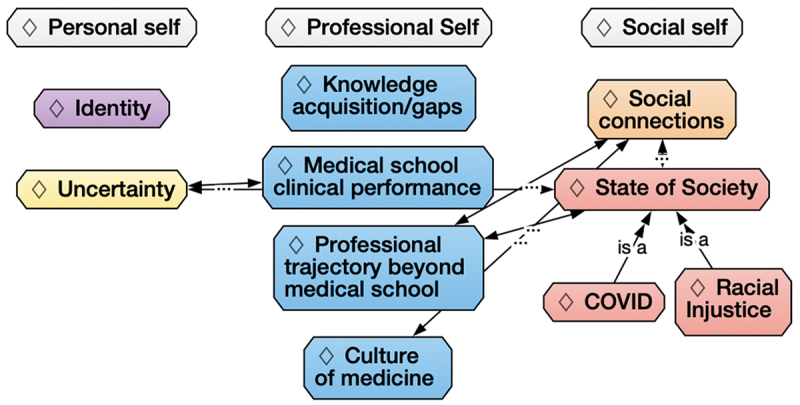
Categorization of codes within schemas of participants’ self-understandings. The personal self related to internal values or life goals, the professional self related to their role as doctors in training, and the social self related to friends, family, and society at large. This figure demonstrates how each of the codes from the initial codebook was categorized into these schemas/themes of self-understanding, and how each was interrelated. Bidirectional arrows indicate codes that often co-occurred in the students’ responses. Colors indicate grouping of codes within spheres of self-understanding. Double arrows indicate subthemes within state of society.

Of 213 enrolled students, 55 (25.8%) consented to having their responses included in the study. Ten codes emerged from participants’ responses: identity, social connections, medical school knowledge acquisition and gaps, medical school clinical performance, professional trajectory beyond medical school, culture of medicine, state of society, COVID-19 pandemic, racial injustice, and uncertainty. Table 1 describes each code with representative quotes. After initial analysis, we mapped these codes into three overlapping schemas of self-understanding: the personal self (related to internal values or life goals), the professional self (related to their role as doctors in training), and the social self (related to friends, family, and society at large), as depicted in Figure 1. This figure demonstrates how each of the codes from the initial codebook was categorized into these realms of self-understanding.

**Table 1.** Explanation of codes with sample quotes. Codes are organized in order of expanding influence, starting at the individual level and ending at the societal level.

Code	Explanation	Sample quote (s)
Identity	Aspirations and concerns related to personal values, life purpose, and life goals, often including an element of self-exploration or introspection.	The owl figure has to hone so many different parts of its identity and talents in order to succeed at the task at hand. Some of these talents may be obscure but are necessary for the one-of-a-kind product that is being made. I hope to bring out the best parts of my character and abilities.
Uncertainty	Perception of ambiguity about what comes next	The field is wide open, with so many possibilities. It looks beautiful, and surely will turn out to be so, but still unpredictable. No one knows what is beyond the expanse, as with my future. This upcoming year is filled with unknowns and uncertainties. It will surely turn out beautiful, but I don’t know exactly what is in store.
Knowledge acquisition/gaps	Reflections about amount of content or process of learning during medical school	I selected this image because to me the wave collapsing over the boat represents the vast amount of knowledge, skills, and other learning that we are expected to attain over the next two years. At times, it seems a bit daunting and like everything around you can come crashing down at any time, kind of like waves crashing over a boat.
Medical school clinical performance	Reflections about how students may perform, fit in, and/or be evaluated during clinical clerkships	I thought this tidal wave image represented how I feel about starting my SubI in July. I feel overwhelmed by returning to a clerkship where I have to perform very well and the expectations are very high for me when I have been absent from the wards for some time and have lost clinical skills.
Professional trajectory beyond medical school	Reflections about the overall career path in medicine after medical school	… I can see I’m in a valley of sorts … no matter how I choose to leave, it will be an uphill climb. This brought to mind the application process, the interview process and the hope to match at my school of choice. There is a lot of beauty and possibility in this painting, and that both excites and scares me.
Culture of medicine	Perceptions about what medicine as a profession might demand and how it might affect identity and relationships.	I think until fairly recently, I had always worried I would have to leave behind my other passions in the pursuit of a medical career … I hadn’t realized it until this year, but this thought had always really bothered me. I had thought I would have to put my outside interests on the back burner and that they would remain in my childhood dreams.
Social connections	Relationships with friends, partners, and/or family	This scene looks like a family sitting down to share dinner together, and it resonates with me because sharing a meal with my friends or family is usually my #1 way to destress and enjoy myself. It’s a universal language of sharing, caring, and relaxing.
State of society	Sentiments about events or challenges impacting society at large. If COVID-19 or Black Lives Matter were specifically mentioned, these were co-coded.	This year more than ever – this has been, and I envision will continue to be, one of the most dysfunctional years of our lifetime. It’s hard not to feel like everything is falling apart around us – the health and safety of our communities, our trust in the institutions that are supposed to protect us, our plans for the future. And yet, we have to keep living in the midst of that, finding our joy whenever we can.
Racial injustice	Reflections on racial injustice and the Black Lives Matter movement	This picture of two young Black children joyfully enjoying the spray from a fire hydrant, especially with the young Black boy positioned in front, portrays ironies that highlight my concerns for the coming year … Another concern is how we as a society will continue to keep the issues of systemic racism and police brutality at the forefront of our actions, even after people stop talking about these issues on social media or the news stops covering protests.
COVID	Reflections on the COVID-19 pandemic and its myriad effects	As a fourth year medical student, over the past several weeks I have been spending a lot of time thinking about a personal future and envisioning residency. I am grateful for all that has changed for me personally in the past three years here at Hopkins and the peaceful landscape of the painting seems emotionally similar to my perspective in that sense. At the same time, I have also been feeling guilt and sadness for all of those who are losing loved ones to COVID.

### The personal self

Personal identity emerged as the most frequent theme during analysis of participants’ narrative reflections. Reflections focused on personal goals, multiplicity of the self, and desire to stay true to oneself. The images that participants selected to accompany these reflections often depicted individual figures in contemplation or concentration, or in moments of connection or spontaneity.

Several participants reflected on aspirations for personal growth, expressing hopes of gaining wisdom and confidence while maintaining a sense of joy and connection. Often, participants mentioned these aspirational qualities in the context of seeking a sense of balance between their work and personal lives. Reflecting upon an image of a human-avian hybrid, one participant wrote, ‘*I hope that one day I will feel like this creature as I study medicine. She seems serene, confident, and in control, even though there are many things going on around her. She seems to be both working hard yet also enjoying life – a balance that I strive to strike every day*.’

**Figure 2.**
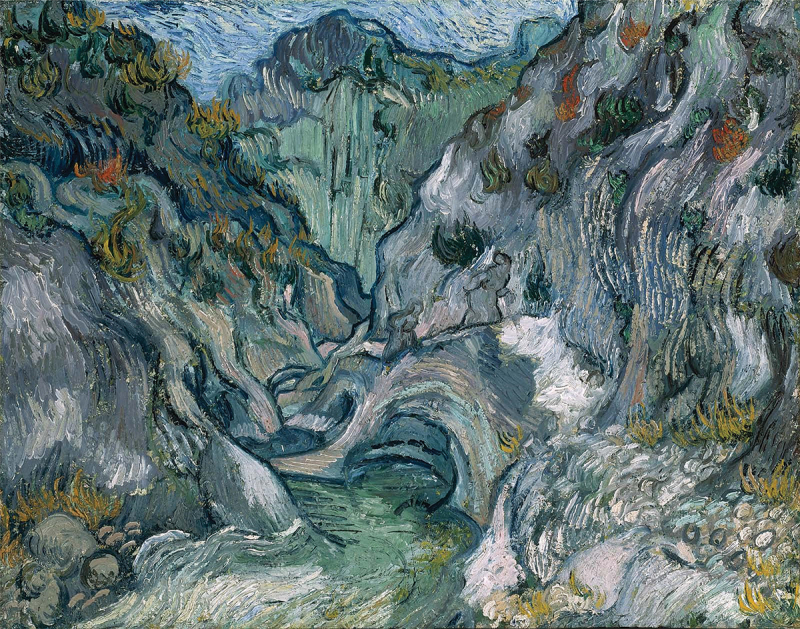
Ravine, Vincent van Gogh, 1889.

Participants also emphasized the composite nature of identity. Considering an image of a series of women with entangled braids, one wrote, ‘*I sometimes feel like there are multiples of myself which are all trying to do something different but these get tangled with one another sometimes*.’ This participant also connected this idea with resiliency, writing about how they hoped their ‘*tangles*’ would withstand the stresses of clinical rotations. Others focused on finding or maintaining their sense of self, humanity, and vitality in times of chaos. One participant chose the image *Ravine* by Vincent van Gogh to represent the uncertainty and ‘*amorphous landscape*’ of the current times (Figure 2). Another described the value of connections in keeping oneself feeling ‘*human*,’ while others emphasized the value of staying true to one’s convictions and to their own ‘*unique identity*.’

### The professional self

Participants also reflected upon their professional selves, specifically around 1) knowledge acquisition, 2) clinical performance, 3) professional trajectory, and 4) overall culture of medicine.

#### Medical school knowledge acquisition

Some participants described concerns about the sheer amount of knowledge they are expected to learn in medical school. These participants selected images that evoked analogies of the ‘*fire hose*,’ the ‘*flood*,’ *‘the infinity*,’ or ‘*waves crashing*.’ When reflecting on knowledge expectations, they invoked terms such as ‘*daunting*’ and ‘*endur[ing] the deluge*.’ These concerns accompanied worries about the pandemic’s effect on their learning, including reduced clerkship length or reliance on virtual experiences, and whether their education would be negatively impacted.

#### Medical student clinical performance

Concern about doing well on clinical rotations also surfaced as an independent theme. Participants wrote about not wanting to burn out. They described fears of lost knowledge due to pandemic delays or concerns about being forgotten about or ignored by clinical team members. One participant mentioned ‘*either staying on or falling off a bucking horse speaks to my concern regarding my ability to keep my head above water in the coming months and that it will likely be a constant battle requiring focus and persistence*.’ Some shared their fears of failure, whether due to being revealed as an ‘*imposter*’ or being too vulnerable or exposed due to the experiences they will encounter in clinical interactions. One participant wrote: ‘*The center shell is almost stripped down to its skeleton and this resonated with me in that of the many other reactions I’ll have to being on the wards, represented by the other shells present, I feel that my emotions will be laid bare by the experiences I will go through and the patients I interact with*.’

In contrast to concerns about performance, participants’ reflections also suggested hope for a positive outcome, with recognition that they might change during the process: ‘*My attitude for most new clerkships now is that I know I’ll never be completely ready so I’ll just make the leap of faith and it’ll probably work out*.’

#### Professional trajectory

Many participants also considered the duration of medical training and reflected on where they were in their journey. Participants viewed medical school as the beginning of a lengthy process. One participant acknowledged the arduous process that still remains, writing, ‘*I can see I’m in a valley of sorts … no matter how I choose to leave, it will be an uphill climb. This brought to mind the application process, the interview process and the hope to match at my school of choice. There is a lot of beauty and possibility in this painting, and that both excites and scares me*.’

Participants expressed concerns about how the COVID-19 pandemic affected their future training. One participant noted that the move to virtual application cycles made them worried about how they would identify the residency training program that was the best fit. Another expressed concern that their personal and professional ‘*path*’ no longer seems as certain as it previously had.

#### Culture of medicine

The final subtheme, within the professional self theme, pertained to culture of medicine. Some participants expressed concerns including being myopic and losing perspective, being stuck in a monotonous routine, or missing the big picture (e.g., ‘*George Floyd. The world is more than medicine*’). Others reflected that, although medicine is a healing profession, the system can be a machine that ignores the welfare of its students, providers, and patients.

Some participants expressed concern that medicine requires uniformity and strips away doctors’ individuality or personal pursuits. One wrote that they always thought a career in medicine would prevent them from keeping other interests alive, but described being heartened by the experiences of a mentor who modeled the marriage of personal interest with one’s medical career. The participant explained: ‘ *… I finally feel like I can include all parts of myself into my future career, and I find it so, so exciting!*’

### The social self

Participants reflected on their sense of self in relation to other individuals and to society at large. Many wrote about the positive effects of relationships on their well-being as a medical student and as a member of society. They shared how friends and family brought them joy, respite, and spiritual rejuvenation, reflecting on how social connections help buffer against personal uncertainty. They also described challenges to relationships posed by medical education, either by virtue of how busy it is or how geographically distant from home it is. Other participants wrote about how the pandemic has affected their relationships, one mourning the difficulties of staying connected while socially distanced, while another mentioned hope for finding a ‘*new normal*.’

Some participants made broad references to the state of society without explicitly invoking COVID-19 or racial injustice. These references tended to relate to the need to fight for justice and maintain hope: ‘*One could draw parallels to our society (fishermen) facing off great injustices and challenges (vs. wave) with a calmer future in the distance (Mt. Fuji) in this work. However, I think this work brings me peace when thinking how people found beauty even in the forces of nature that constantly threatened their nation*.’ In other cases, participants reflected on systemic racism and societal response, which we coded under the racial injustice theme. Participants specifically mentioned racial injustice as a challenge for the future, for themselves and their society. For some participants, the theme of racial injustice heightened their own concerns about faith and purpose (‘*is there a reason for all this?*’) or reminded them to look beyond the insular world of medicine or further in the future, if media coverage eventually fades. Participants described racism-related challenges as a personal challenge to address and an impetus for making change on a broader society level, along with concerns about how they will fit this calling into their medical professional career.

Amidst these societal reckonings, some participants wrote that they sought comfort amidst images that evoked a sense of community, harmony, and peace. One participant commented: ‘*Just feels nice to see a comforting image of Black people especially during these difficult times of societal upheaval*.’ Another took inspiration from an image showing a diverse group of people who were physically connected, writing, ‘*The image shows a diverse group of women inexplicably tied together, though trying to separate. The need to separate appears urgent given the falling objects. This reminded me of the social unrest ongoing in our country. On the other hand, by nature of the hair being tied together, it seems to be forming a net upon which the objects could safely land*.’

## Discussion

In our analysis of medical students’ reflections on moments of transition through the lens of visual art, we found that participants frequently entered into self-inquiry on various aspects of their identity. Students experienced this visual arts-mediated reflection during a pivotal time of transition, in both microscopic and macroscopic planes. Transitions experienced included progressing from preclinical to clinical years or from medical school to residency, as well as broader societal changes during a global pandemic and social justice movements. Our exploratory study demonstrates a cross-section of attitudes towards identity during transitions in medical education and suggests that the use of visual art in reflection among health professions education is both feasible and potentially effective in encouraging authentic reflection.

Our analysis of participants’ reflections on identity aligns well with what has been previously described in the literature: that identity comprises personal, relational, and collective spheres [14]. Cruess et al describe how ‘*the identity of an individual at any moment represents the sum of the influences impacting these three domains*’ [15]. The personal domain comprises ‘*personal characteristics, self-chosen or mandated commitments, beliefs about one’s self, and the impact of multiple life experiences*’ [15]. The relational domain includes *‘the influence on identity of significant individuals, such as family members, friends, mentors, and coworkers*’ [15]. Finally, the collective domain represents ‘*the impact of the social groups to which an individual belongs or wishes to join*’ [15]. This approach also resonates with the ring theory of personhood, which as defined by Sarraf-Yazdi et al encapsulates the innate, the individual, the relational, and the societal [16]. That our schemas often overlapped with one another is also consistent with conceptual literature on personal and professional identity; the rings of personhood, for instance, are described as being ‘*interconnected*’, and identity formation, especially in professional settings, is ‘*complex, non-linear, and fluid*’ [16].

Within the individual domain, participants explored their aspirations, reflected on the many ‘*selves*’ comprising their individual identity, and expressed a desire to stay true to this identity during moments of change and transition. Participants’ statements, within the relational domain, described both the value of and threats to personal connections. These encompassed two types of collective domains: both the professional domain and the social. Reflections on medical school education included excitement or anticipation, but also distress about the rigors of the process and the impacts of the uncertainty wrought by the disruption of traditional medical education due to the pandemic. Others described the identity dissonance that may occur over the course of medical education, including the perceived threat of enforced uniformity, the difficulty of staying ‘*true*’ to oneself as identities are de- and re-constructed during training, and the tension between expectation and reality within the culture of medicine [17]. Participants also considered their hopes and concerns within the broader context of social change and upheaval, with some grappling with their roles and relationships with societal injustice. Our findings provide both a snapshot of a unique and historic moment in medical education, as well as a cross-section of medical students’ perceptions of transitions in medical education and training in general.

Our study specifically revealed that reflections on moments of change or transition may lead to self-inquiry on identity. Indeed, it is well documented in the literature that reflection is key to identity formation. Reflection is a core component of the process of socialization necessary to successful transformation of personal and professional identities as students transition to healthcare professionals [15,16]. Creating a ‘*pedagogical space*’ where students can make sense of their experiences in the context of personal values and broader professional culture can lend itself to an understanding of their identity and a potential integration of personal beliefs and professional attributes [15,17]. Through individual reflection, one considers both clinical and nonclinical aspects of one’s life, including personal clinical experiences, interactions with clinicians, and perspectives from friends and family. This reflection is ideally facilitated by role models and mentors, with opportunities for discussion with colleagues [14,15,17–19].

Amidst the demands of medical education, it may be challenging to provide protected time for reflection, to ensure all learners have an opportunity to share, and to create a safe space where learners feel comfortable with potentially being vulnerable. Our hybrid, visual arts-mediated approach may represent some solutions to such barriers. Reflection occurred asynchronously, permitting students to engage in the reflective activity at their convenience. Interactional sharing of reflections subsequently occurred, through sharing of deidentified narrative reflections, permitting those who may not feel comfortable with speaking aloud to still share their voices and peruse their colleagues’ reflections at their leisure. Although we did not examine the impact of our activity on learners, visual art has been established as a successful tool for deeper, and potentially more authentic and open, introspection [8–10].

In their narrative reflections, participants made specific references to elements of their selected works of art, suggesting that the images themselves served as inspiration, or visual cues, to then launch them into further self-exploration. This was strengthened by the established role of art as a ‘third thing,’ or a reflective trigger or conversational mediator, which creates a safe space for revelation and reflection. Gaufberg et al described how visual arts-based stimuli for reflection in the health professions allows for metaphors to be accessed indirectly through images that do not require traditional medical expertise [8]. In this way, the artworks create a sense of emotional and psychological safety, encourage exploration of assumptions and beliefs, and flatten traditional hierarchies that may otherwise inhibit sharing of perspectives. In our experience, we found that the use of art in combination with a written prompt creates synergy that stimulates further thought and helps students delve into themes that they may not have explored when provided with either a work of art or a written prompt alone. This visual arts-assisted approach to reflection holds promise in health professions education. Medical educators are already finding creative ways to engage with the arts to deepen reflection, including collaborations with art museums, and our virtual adaptation of such approaches demonstrates that they can also be performed successfully outside the setting of the museum [20].

Our study has certain limitations. It occurred at a single site, for a two-month period, at a very particular time during our modern history. These aspects may limit generalizability, and similar themes may not have arisen if the activity were conducted at a different time. However, our conclusions on reflection and identity are congruent with previous literature. Additionally, we believe that, conversely, this may also represent a strength of our study since it is providing a unique snapshot of a historic moment in medical education. It is possible that the 25% of participants opting to include their responses may have been subject to self-selection bias; however, the post-course consent process may have increased the authenticity of responses as students would have had no concerns at the time of reflection about the inclusion of their responses in a research study. Additionally, we did not evaluate any outcomes of participation in this exercise.

This topic represents fertile ground for study. Our study would have benefited from a comparison arm between subsequent or preceding medical student classes, as well as across institutions. Additionally, we believe this exercise in reflection could be longitudinally studied at various transition points throughout training, which may bring to light additional themes or deepen our understanding of how reflection and identity evolve across time. Future studies should also consider exploring these reflections within the broader context of how students operationalize identity.

One continued challenge remains finding scheduled time to ensure opportunities for guided clinical reflection. Participants in our cohort had protected time to engage in reflection during a required course. Additionally, not all training sites will have faculty with experience in arts-based health professions education; however, freely-available resources exist for faculty development [21].

## Conclusions

In this qualitative analysis of medical students’ visual arts-mediated written reflections during a unique moment of transition, we found that students often linked their hopes and concerns for their new clinical roles with the evolution of their own identities. The act of reflection is often described as a critical part of professional identity formation and professional development, and it may be especially crucial at moments of transition [22]. A visual arts-mediated approach demonstrates promise in prompting reflection on self and identity in personal, professional, and social spheres. Medical schools should consider integrating such low-cost, portable, and time-efficient approaches to reflection into their curriculum. Though additional investigation is needed to ascertain the ultimate impacts of such methods, the creation of time and safe space for reflection may help build the foundation for continued personal development along a clinician’s career. As threats to trainees’ well-being and resiliency continue to grow, this type of reflection becomes ever more impactful, with the potential to facilitate individual and community growth.
